# Community-Based Interventions in Prepared-Food Sources: A Systematic Review

**DOI:** 10.5888/pcd10.130073

**Published:** 2013-10-31

**Authors:** Joel Gittelsohn, Seung Hee Lee-Kwan, Benjamin Batorsky

**Affiliations:** Author Affiliations: Seung Hee Lee-Kwan, Benjamin Batorsky, Center for Human Nutrition, Johns Hopkins Bloomberg School of Public Health, Baltimore, Maryland.

## Abstract

**Introduction:**

Food purchased from prepared-food sources has become a major part of the American diet and is linked to increased rates of chronic disease. Many interventions targeting prepared-food sources have been initiated with the goal of promoting healthful options. The objective of this study was to provide a systematic review of interventions in prepared-food sources in community settings.

**Methods:**

We used PubMed and Google Scholar and identified 13 interventions that met these criteria: 1) focused on prepared-food sources in public community settings, 2) used an impact evaluation, 3) had written documentation, and 4) took place after 1990. We conducted interviews with intervention staff to obtain additional information. Reviewers extracted and reported data in table format to ensure comparability.

**Results:**

Interventions mostly targeted an urban population, predominantly white, in a range of income levels. The most common framework used was social marketing theory. Most interventions used a nonexperimental design. All made use of signage and menu labeling to promote healthful food options. Several promoted more healthful cooking methods; only one introduced new healthful menu options. Levels of feasibility and sustainability were high; sales results showed increased purchasing of healthful options. Measures among consumers were limited but in many cases showed improved awareness and frequency of purchase of promoted foods.

**Conclusion:**

Interventions in prepared-food sources show initial promising results at the store level. Future studies should focus on improved study designs, expanding intervention strategies beyond signage and assessing impact among consumers.

## Introduction

Obesity is a multifactorial disease that has many contributing factors, including the food environment. Food environments in which energy-dense prepared foods (ready-to-eat foods that can be eaten outside the home or brought back or delivered to the home to eat) are readily available are associated with the increasing prevalence of obesity (1–3). As a consequence, environmental interventions may be more cost-effective, sustainable, and successful at reaching a large population than individual interventions (4). Today, Americans spend about half of their food dollars eating out (5,6) compared with 25% in 1955 (7). Americans are expected to spend $660.5 billion in 2013 on eating out, a 15-fold increase from 1970 (adjusted to current dollars) (7). The total energy intake per capita increased on average by 570 kcal between 1977–78 and 2003–06; this increase has been attributed to greater portion sizes and a greater frequency of eating out (8). Although prepared foods are generally more costly than foods prepared at home, people who have low incomes consume them at high rates (9–11). Prepared-food sources may be an important venue for efforts to reduce obesity and risks for other chronic diseases. In the past several decades, multiple interventions were conducted in venues that provide prepared foods. One review (12) showed that interventions had success in private prepared-food–source environments, such as worksite and college cafeterias. However, no systematic reviews examined prepared-food source interventions in public community settings such as carryout, fast-food, and sit-down restaurants, even though prepared-food sources are more numerous in public settings than in private settings, and low-income individuals may have little access to private settings, which may offer more healthful options. The objective of this study was to systematically review community-based interventions in prepared-food sources that aimed to increase access to and consumption of healthful foods. 

## Methods

This systematic review was designed to identify interventions in public prepared-food sources; to present data on strategy, study design, evaluation, process indicators, and impact for each intervention identified; and to suggest next steps in research, practice, and policy. We followed the Preferred Reporting Items for Systematic Reviews and Meta-Analyses (PRISMA) guidelines, which provide 27 checklist items that aim to standardize the structure of meta-analyses and ensure comprehensive reporting (13).

### Data sources

From September 2011 through January 2013, we used PubMed and Google Scholar to search peer-reviewed journals and Google to search “gray” literature for articles on interventions in prepared-food sources. Gray literature included newsletters, published (but not peer-reviewed) articles, policy briefs or reports, published trial materials, and conference presentations. Search terms for each engine included the following: “restaurant intervention,” “nutrition AND intervention,” “fast-food AND intervention,” “prepared food intervention,” “point of purchase food,” “healthy food choices,” and “restaurant health food choices.”

### Study selection

We initially identified 35 potential interventions. All identified food-source interventions were reviewed for inclusion according to the following criteria: 1) a focus on prepared-food sources in public community settings such as carryout, fast-food, and sit-down restaurants (although other intervention components could be included as part of the intervention); 2) a completed impact evaluation (eg, pre–post assessment, use of a comparison group, exposure assessment with or without a comparison group); 3) some form of written documentation (eg, peer-reviewed journal article, newsletter article, other published article, policy brief or report, published trial material, a project’s own website or conference presentation) that included a description of the implemented intervention and evaluation findings; and 4) an intervention start date after 1990. Of the original 35 interventions, 19 met these inclusion criteria.

For 4 of the 19 interventions, only website information was available, and because we were unable to obtain further information, these 4 were also excluded from our review. Of the 15 remaining, 2 interventions lacked information on study findings and were therefore excluded. These exclusions left 13 interventions for review, data extraction, and analysis.

### Data extraction

The 2 primary reviewers (S.L.K., B.B.) independently extracted and analyzed data by reviewing all documents. The secondary reviewer (J.G.) developed the system for extracting data and coding variables. The secondary reviewer also resolved discrepancies noted by the 2 primary reviewers and identified and adjudicated other discrepancies that might affect reliability and analysis (Appendix).

Primary reviewers were instructed to extract data for each variable and to organize data using the intervention as the unit of analysis. We attempted to contact a representative of each intervention to obtain more information. Seven of 13 intervention managers or lead researchers participated in semistructured telephone interviews or e-mail communications (or both) designed to gather additional information needed to complete the data tables and to resolve any inconsistencies. One intervention (Baltimore Healthy Carryouts) was implemented by our team. After the interventions were reviewed, we summarized data in 3 tables: background and intervention approach, evaluation methods used, and study findings. Within each table, we grouped restaurants into 4 categories according to similar characteristics: specialty restaurants, chain restaurants, small local restaurants, and mixed types of restaurants (both local and chain) that did not share characteristics with other restaurants or whose characteristics were not well defined. The primary reviewers organized the information about each study into these tables. The secondary reviewer confirmed data accuracy by using initial review findings, e-mail correspondence, interview transcripts, and extraction and reporting guidelines.

We implemented previously used techniques (14) for analytic assessment of the selected interventions, such as identifying a standard set of quality criteria (eg, randomization and use of control groups) and reporting on the impact among prepared-food sources and consumers. Because of the heterogeneity of outcome data, which did not permit us to calculate summary estimates of impact, meta-analytic techniques were not used.

## Results

Of the 13 interventions (15–49) that met the inclusion criteria, 12 interventions (15–18,21,22,24–35,37–41,45–48) were described in peer-reviewed publications. Personal communications (e-mail correspondence, telephone interviews) (n = 11) (15–27,30,31,37–49), informal documentation (reports, intervention materials) (n = 5) (19,20,26,43,44,49), and websites (n = 5) (23,36,42,43,49) were also sources of information (Table 1a, 1b, 1c, 1d).

**Table 1a:** Key Components of Interventions on Community-based Prepared-Food Sources: Interventions Conducted in Specialty Restaurants^a^

Characteristic	Baltimore Healthy Carryouts (15–23)	Good for You (24)	Steps to a Healthier Salinas (25,26)	Horgen and Brownell 2002 (27)
**Data sources**	Peer-reviewed articles; conference presentation; website	Peer-reviewed article; e-mail correspondence	Peer-reviewed articles; interview; e-mail correspondence; intervention materials	Peer-reviewed article; interview
**Target population**	Baltimore; African American; low-income; urban	Minnesota; Target store customers	Salinas, California; low-income; mostly Mexican American	New Haven, Connecticut; delicatessen customers; mostly white, upper-middle class
**Model/theory**	Social cognitive theory; Social marketing	Not specified	Socioecological model; asset-based community development	Matching model; health belief model
**Goals**	Awareness; availability; affordability; consumption	Awareness; consumption	Awareness; consumption; availability	Awareness; consumption; affordability
**Types of prepared-food sources**	Carryout restaurants serving mainly deep-fried foods	Cafeteria in large general merchandise/department store	Mexican-style restaurants	Delicatessen
**Food targeted in intervention**	Low-fat, low-cholesterol entrées, sides, and beverages	15 Low-fat items on menu	Healthier preparations for dishes; replacement of lard with vegetable oil for cooking	Low-fat meals and sides
**Intervention strategies**				
**Signage**	Menu board; menu labeling; point-of-purchase posters	Menu labeling; point-of-purchase materials (table tents, tray liners)	Point-of-purchase materials (logo on menu and signage)	Price reduction messages; point-of purchase materials (signs, posters)
**Increased availability of healthful foods**	Fresh fruits, yogurts, healthful sides, and grilled chicken sandwich	None	Healthful preparation strategies; fresh fruit juices	None
**Pricing**	Reduced-price combination meals	None	Coupons (10% discount)	20%–30% price reduction of low-fat items
**Community components**	None	None	None	None
**Other**	None	None	Healthy Nutrition Tool kit; give-aways	None
**Formative research**	Semi-structured interview with customers (N = 50) and owners (N = 12); focus groups, conjoint analysis (N = 50)	Customer survey of interest in buying lower-fat foods at Target Food Avenue	Surveys of local taquerias; work with owners to identify intervention strategies	Informal discussions with restaurant managers and staff

a Includes restaurants, such as carryouts, taquerias, and delicatessen cafés, that focused on specialty foods; it excludes chain fast-food restaurants.

**Table 1b:** Key Components of Interventions on Community-based Prepared-Food Sources: Interventions Conducted in Chain Restaurants

Characteristic	Coeur en Santé St-Henri (28,29)	TrEAT Yourself Well (30)	Tandon et al 2011 (31)
**Data sources**	Peer-reviewed article	Peer-reviewed article; e-mail correspondence	Peer-reviewed article; e-mail correspondence
**Target population**	Montreal; restaurant customers; low-income	San Diego area; restaurant customers; mostly white	Seattle and San Diego; children aged 6–11 years and parents
**Model/Theory**	Social learning theory	Theory of reasoned action; social marketing	None specified
**Goal**	Awareness; consumption	Awareness; consumption	Awareness; consumption
**Types of prepared-food sources**	Fast-food restaurant and family-style restaurant	4 chain restaurants	Chain restaurants
**Food targeted in intervention**	Low-fat, high-fiber items on menus	Low-fat menu items containing fruits and vegetables	Chain restaurant foods
**Intervention strategies**			
**Signage**	Menu labeling	Point-of-purchase materials (table tents, posters)	Menu labeling
**Increased availability of healthful foods**	Recipe modification to increase fiber or decrease fat	None	None
**Pricing**	None	Discount cards	None
**Community components**	Media (newspaper, telephone, leaflets)	Community events and food tasting; media (television, magazines, newspaper)	None
**Other**	None	Wait-staff incentives	None
**Formative research**	Community informants identify popular restaurants; dietitian review of menu options via interviews with kitchen staff supervisor and suppliers	None	None

**Table 1c:** Key Components of Interventions on Community-based Prepared-Food Sources: Interventions Conducted in Small Local Restaurants^a^

Characteristic	Shape Up Somerville (32–36)	Smart Menu Program (37,38)	The Healthy Options Program (39–42)
**Data sources**	Peer-reviewed articles; website	Peer-reviewed articles; e-mail correspondence	Peer-reviewed article; website; e-mail correspondence
**Target population**	Somerville, Massachusetts; restaurant customers; students in 1st–3rd grade	Tacoma-Pierce County, Washington; restaurant customers; mostly white	Rural Iowa; mostly white
**Model/Theory**	Community-based participatory research	Diffusion of innovations	Social cognitive theory
**Goal**	Awareness; availability	Awareness; consumption	Awareness; consumption
**Types of prepared-food sources**	Various participating restaurants	Various participating locally owned restaurants	Various locally owned restaurants
**Food targeted in interventions**	Half-size portions; low-fat milk or water	Meal and dessert items	Low-fat dressings, milk, meat, sides, breakfast items, desserts, whole-wheat bread
**Intervention strategies**			
**Signage**	Menu labeling; point-of-purchase materials (logo and signs, menu inserts)	Menu labeling	Point-of-purchase materials (table placard, poster)
**Increased availability of healthful foods**	Half-sized portions of entrées; fruit and vegetable side dishes; low-fat milk or water	Encouragement of addition of healthful items, preparations, smaller portion sizes	None
**Pricing**	None	None	None
**Community components**	Improved walkability and environmental policies; community advocates established; farmers market program; newspaper ads	Media (newsletters, newspapers, websites)	Media (newspaper)
**Other**	None	Free menu analysis	None
**Formative research**	Meetings, focus groups, and interviews with owners/managers; advisory council; approval criteria were refined with feedbacks from restaurant owners and managers; unable to complete 3 focus groups with restaurants	Menu labeling literature review; consulted food industry representative and health advisory group; no formal formative research phase.	Pilot survey to indicate customer preferences; presented to owners

a Includes small, locally owned “mom-and-pop” establishments that include but are not limited to take-out and sit-down restaurants and restaurants that focused on specialty foods; it excludes chain restaurants.

**Table 1d:** Key Components of Interventions on Community-based Prepared-Food Sources: Interventions Conducted in Mixed Types of Restaurants^a^

Characteristic	Healthy Howard Initiative (43)	Healthy Restaurant Program (44–46)	Winners Circle Healthy Dining Program (47–49)
**Data sources**	Interview; website; printed materials; e-mail correspondence	Peer-reviewed article; research report; interview	Peer-reviewed article; conference presentation; website; e-mail correspondence
**Target population**	Howard County, Maryland; restaurant customers	Seoul, South Korea; restaurant customers	65 North Carolina counties; mixed race/ethnicity
**Model/theory**	Community-based environmental change initiative	Community capacity analysis	Social marketing; community-based environmental-change initiative
**Goal**	Awareness; consumption	Awareness; consumption	Awareness; consumption
**Types of prepared-food sources**	Various participating restaurants	Various participating restaurants	Various participating restaurants
**Food targeted in interventions**	Healthful entrées that meet nutritional criteria	Healthful menu items that meet nutritional criteria	Meals, side items, snacks and beverages good for “heart health”
**Intervention strategies**			
**Signage**	Menu labeling; point-of-purchase materials (window decal and certificate, nutrition sheets)	Menu labeling; point-of-purchase materials (photos, menu boards, posters, logo on restaurant)	Point-of-purchase materials (logo on menu, promoted items)
**Increased availability of healthful foods**	Trans fat-free; healthful menu options	Foods that meet nutritional standards	None
**Pricing**	Discounts (planned, not yet implemented)	None	None
**Community components**	Media (newspaper, magazine, websites); community events	Media (newspaper, website); community events	Media (billboards, television)
**Other**	Comply with the “Clean Indoor Air Act”; pass food inspections; allergen labeling on menu	None	Nutritional information in booklets or brochures in some locations
**Formative research**	None	Survey of customers and restaurant workers for information on how to design intervention; focus groups with restaurant workers; in-depth interviews with consumers	Pilot in 2 North Carolina counties; intercept interviews

a Includes restaurants (both local and chain) that did not share characteristics with other intervention restaurants or whose characteristics were not well defined.

### Target populations

Most interventions (n = 9) took place in urban settings; 3 interventions focused on mixed urban and rural populations, and one focused on rural settings. Interventions were conducted in various regions of the United States: 4 in the Northeast, 4 in the West, 2 in the Midwest, and 1 in the Southeast. Only 2 interventions took place outside the United States (Table 1a, 1b, 1c, 1d). The interventions targeted a range of consumer income levels: 3 interventions (Baltimore Healthy Carryouts [15–23], Steps to a Healthier Salinas [25,26], and Coeur en Santé [28,29] worked in low-income areas, Horgen and Brownell (27) worked in middle- to high-income areas, and the rest did not identify the income level of the targeted population. The race/ethnicity of consumers in the interventions was generally mixed: 5 populations were predominantly white; 1, Hispanic; 1, African American; 1, Korean; and 1, mixed; 4 interventions did not specify the race/ethnicity of the targeted population.

### Behavior-change theory

Ten interventions explicitly stated the theoretical frameworks that guided their design (15–23,25–30,32–49). The theory most commonly identified was social marketing (n = 3), used by Winners Circle (47–49), TrEAT Yourself Well (30), and Baltimore Healthy Carryouts (15–23). No other theory was identified by more than 1 trial; however, an emphasis on changing the food environment was mentioned in Baltimore Healthy Carryouts (15–23), Horgen and Brownell (27), and Shape Up Somerville (32–36).

### Intervention goals

Overall, the 13 interventions shared similar goals. All sought to promote more healthful menu items at prepared-food sources with the associated goal of increasing sales of these foods. Some interventions had additional goals. The larger goal of Shape Up Somerville (32–36) was to reduce childhood obesity, and their restaurant intervention (33) was viewed as an environmental component of that work. The additional goal of Baltimore Healthy Carryouts (15–23) was to assess the feasibility of a carryout intervention. Other projects stated that they sought to achieve their overall goals by specific means, such as menu labeling (Tandon et al [31] and Smart Menu program [37,38]) and price changes (Horgen and Brownell [27]).

### Formative research

Three interventions (30,31,43) conducted no formative research before designing and implementing their program. Of the 10 that conducted formative research, 2 reported “informal” research (27,37,38), consisting mainly of literature reviews and discussions with a small number of stakeholders. The remaining 8 interventions did more extensive formative research, including qualitative (eg, focus groups, in-depth interviews) and quantitative (eg, surveys) data collection. Some interventions published separate reports on their formative research (15,18,21,22,25,28,41,45,46,49).

### Intervention strategies

#### Food promotion

All interventions focused on identifying and promoting existing healthful food options at prepared-food sources as the primary intervention strategy. Five interventions identified specific nutritional criteria for a food to be considered healthful (Coeur en Santé [28,29], TrEAT Yourself Well [30], Tandon et al [31], Healthy Howard [43], and Healthy Restaurant [44]). Only 1 intervention (Baltimore Healthy Carryouts [15–23]) introduced new healthful options: lower-fat side dishes and grilled chicken. All interventions promoted healthful entrées, but 5 interventions also promoted healthful snacks and side dishes: for example, Winners Circle (47–49), Shape Up Somerville (32–36), Baltimore Healthy Carryouts (15–23).

#### Food preparation or portion size

Six interventions worked with restaurant owners and staff to develop more healthful recipes, usually by using lower-fat cooking methods (15–23,25,26,28,29,32–38,44–46). Two interventions, Shape Up Somerville (32–36) and the Smart Menu program (37,38), aimed to decrease the portion size of foods served.

#### Signage and menus

All 13 interventions used some form of signage to promote more healthful options. Most interventions (n = 10) used some form of menu labeling, usually a symbol to indicate more healthful choices. Baltimore Healthy Carryouts (15–23) redesigned carryout menus in low-income areas of Baltimore to emphasize more healthful choices. Other forms of signage included posters (n = 4) and menu inserts or tablemats (n = 4).

#### Pricing or cost reduction

Four interventions sought to reduce the consumers’ cost for the more healthful items. Steps to a Healthier Salinas (25,26) and TrEAT Yourself (30) included coupons or discount cards; Baltimore Healthy Carryouts (15–23) and Horgen and Brownell (27) implemented a price reduction. Baltimore Healthy Carryouts focused on price reductions for combination meals as a strategy (19,22).

#### Community promotion

Four interventions did not promote their programs outside the prepared-food sources or in the general community. Of the 7 interventions that promoted their programs widely, a variety of approaches were used, including newspaper advertisements (n = 5), promotion at community events (n = 3), and leaflets and newsletters (n = 3). These promotions were intended to increase awareness of the program and to direct consumers to prepared-food sources participating in the intervention.

### Study design

Most interventions were nonexperimental interventions in which participation by the prepared-food sources was voluntary (n = 10). Two interventions had a quasi-experimental design: Shape up Somerville (32–36) and TrEAT Yourself Well (30). Baltimore Healthy Carryouts (15–23) used a true experimental design, with random assignment to treatment. Nearly half (n = 6) of the interventions (24,27,37–42,44–46) had pre–post assessment at the prepared-food source level with no comparison group (Table 2a, 2b, 2c, 2d).

**Table 2a:** Evaluation Methods Used by Interventions on Prepared-Food Sources, by Type of Intervention: Interventions Conducted in Specialty Restaurants^a^

Characteristic	Baltimore Healthy Carryouts (15–23)	Good for You (24)	Steps to a Healthier Salinas (25,26)	Horgen and Brownell 2002 (27)
**Study design**	Experimental design; pre–post assessment (n = 8)	Nonexperimental; pre–post sales analysis, broken down by quarter (n = 7)	Nonexperimental; no pre–post assessment; intervention trial, voluntary participation; no comparison group (n = 16)	Nonexperimental; pre–post assessment (n = 1)
**Feasibility assessment measures^b^**	Informal observation; staff reports; interviews with carryout owners or staff	Launched simultaneously in all Target Food Avenue restaurants; not assessed at individual store level	Assessments, discussion with health educators	Informal visits, daily check-in
**Process evaluation measures^c^**	Direct observation	None	Surveys with store owners; informal observation	Informal visits, daily check-in
**Prepared-food source impact measures**	Sales	Sales	None	Sales
**Consumer impact measures^d^**	Purchasing; awareness; self-reported body mass index	None	Modified Behavioral Risk Factor Surveillance System	Behavior

a Includes restaurants, such as carryouts, taquerias, and delicatessen cafés, that focused on specialty foods; it excludes chain fast-food restaurants.

b Feasibility assessment measures include acceptability, operability, and perceived sustainability.

c Process evaluation measures include dose, reach, and fidelity, which indicate how well the program was implemented according to plan.

d Consumer impact measures included psychosocial, behavioral, and health outcomes.

**Table 2b:** Evaluation Methods Used by Interventions on Prepared-Food Sources, by Type of Intervention: Interventions Conducted in Chain Restaurants

Characteristic	Coeur en Santé St-Henri (28,29)	TrEAT Yourself Well (30)	Tandon et al 2011 (31)
**Study design**	Nonexperimental; no pre–post assessment (n = 2)	Quasi-experimental; no pre–post assessment; comparison regions (n = 4)	Pre–post assessment; comparison counties
**Feasibility assessment measures^a^**	None	None	None specified
**Process evaluation measures^b^**	None	None	None specified
**Prepared-food source impact measures**	None	None	None
**Consumer impact measures^c^**	Purchasing; attitudes	Awareness; attitudes	Awareness; behavior (calories consumed)

a Feasibility assessment measures include acceptability, operability, and perceived sustainability.

b Process evaluation measures include dose, reach, and fidelity, which indicate how well the program was implemented according to plan.

c Consumer impact measures included psychosocial, behavioral, and health outcomes.

**Table 2c:** Evaluation Methods Used by Interventions on Prepared-Food Sources, by Type of Intervention: Interventions Conducted in Small Local Restaurants^a^

Characteristic	Shape Up Somerville (32–36)	Smart Menu Program (37,38)	The Healthy Options Program (39–42)
**Study design**	Quasi-experimental; nonexperimental for restaurant portion of intervention; intervention trial, voluntary participation (n = 21)	Nonexperimental; pre–post assessment; intervention trial, voluntary participation; no comparison group (n = 6)	Nonexperimental; pre–post assessment (n = 4)
**Feasibility assessment measures^b^**	Environmental change assessment; owners’ compliance and perceived impact	Interviews with restaurant owners or managers	Interviews with owner and staff
**Process evaluation measures^c^**	Extensive process evaluation; participation and adherence to intervention elements	Observation of nutrition information being posted	None
**Prepared-food source impact measures**	Owner survey (menu changes, sales, nutrition awareness)	Sales	Sales
**Consumer impact measures^d^**	None for restaurant intervention; assessment at child and household level (change in body mass index)	Awareness; behavior	Awareness; behavior

a Includes small, locally owned “mom-and-pop” establishments that include but are not limited to take-out and sit-down restaurants and restaurants that focused on specialty foods; it excludes chain restaurants.

b Feasibility assessment measures include acceptability, operability, and perceived sustainability.

c Process evaluation measures include dose, reach, and fidelity, which indicate how well the program was implemented according to plan.

d Consumer impact measures included psychosocial, behavioral, and health outcomes.

**Table 2d:** Evaluation Methods Used by Interventions on Prepared-Food Sources, by Type of Intervention: Interventions Conducted in Mixed Types of Restaurants^a^

Characteristic	Healthy Howard Initiative (43)	Healthy Restaurant Program (44–46)	Winners Circle Healthy Dining Program (47–49)
**Study design**	Nonexperimental design; intervention trial, voluntary participation; no comparison group	Nonexperimental design; pre–post assessment; intervention trial, voluntary participation; no comparison group	Nonexperimental; intervention trial, voluntary participation; cross-sectional survey of community awareness of program; no comparison group
**Feasibility assessment measures^b^**	Restaurant owner or manager survey; focus group; recipe analysis	Interviews with chefs; survey of restaurant managers or staff	Survey of managers
**Process evaluation measures^c^**	Informal observation, telephone communication; annual health inspection; recertification every 2 years	Annual menu analysis; annual observation	Tracked reach and dose using Winner’s Circle team reporting forms; menu review
**Prepared-food source impact measures**	Restaurant owner or manager survey (recall of sales)	Restaurant owner survey	None
**Consumer impact measures^d^**	Psychosocial, behavioral survey	Awareness; attitudes	Awareness

a Includes restaurants (both local and chain) that did not share characteristics with other intervention restaurants or whose characteristics were not well defined.

b Feasibility assessment measures include acceptability, operability, and perceived sustainability.

c Process evaluation measures include dose, reach, and fidelity, which indicate how well the program was implemented according to plan.

d Consumer impact measures included psychosocial, behavioral, and health outcomes.

### Evaluation methods used and key findings

#### Feasibility and process evaluation of interventions

Most interventions (n = 8) collected information on feasibility. In general, this information was in the form of interviews and informal discussions with owners and managers of the prepared-food sources. These same 8 interventions also conducted some form of process evaluation (usually through store visits) to assess, for example, whether signage was displayed and healthful options were available.

Overall, the level of feasibility was moderate to high for intervention implementation (Table 3a, 3b, 3c, 3d). The level of acceptability was generally high among participating food-source owners. Menu labeling was particularly acceptable among multiple interventions. However, no trial assessed program acceptability among consumers.

**Table 3a:** Feasibility, Process, and Impact Results of Interventions on Prepared-Food Sources: Interventions Conducted in Specialty Restaurants^a^

Characteristic	Baltimore Healthy Carryouts (15–23)	Good for You (24)	Steps to a Healthier Salinas (25,26)	Horgen and Brownell 2002 (27)
**Feasibility and process results**	High acceptability and fidelity for Phase 1 and Phase 3; medium acceptability and operability for Phase 2; high dose received	Not assessed	Medium feasibility; medium reach to owners; moderate to high fidelity in terms of changes to menu items	Assessed but results not reported
**Prepared-food source impact results**	Increase in sales of promoted items	Increase in sales of promoted items	Not assessed	Increase in sales of promoted items, especially with price reductions
**Consumer psychosocial impact results**	Not assessed	Not assessed	Assessed but results not reported	Assessed but results not reported because of low response rate
**Consumer behavioral impact results**	Increased purchasing	Not assessed	Not assessed	Not assessed
**Other results**	Community members’ positive response toward the intervention	Seasonality of sales of some foods (salad, frozen yogurt)	Mistrust of health educators by restaurant owners	Health messages not very effective
**Sustainability**	Low implementation cost	Sales of labeled foods remained high	High sustainability: most signage still displayed 4 years later	Went out of business
**Policy results, implications**	Disseminated citywide as a public market carryout strategy “Get Fresh Public Markets”	Supports effectiveness of menu labeling	Considering permits based on healthful food offerings	Subsidies on healthful foods can increase sales

a Includes restaurants, such as carryouts, taquerias, and delicatessen cafés, that focused on specialty foods; it excludes chain fast-food restaurants.

**Table 3b:** Feasibility, Process, and Impact Results of Interventions on Prepared-Food Sources: Interventions Conducted in Chain Restaurants

Characteristic	Coeur en Santé St-Henri (28,29)	TrEAT Yourself Well (30)	Tandon et al 2011 (31)
**Feasibility and process results**	High acceptability among owners and customers	Not assessed	None
**Prepared-food source impact results**	Not assessed	Not assessed	None
**Consumer psychosocial impact results**	Awareness of campaign; intentions to eat healthier	High awareness; beliefs about healthful food	High awareness of nutrition information
**Consumer behavioral impact results**	Increased purchasing in family-style restaurant compared with fast-food restaurant	Increased purchasing of healthful foods	No difference in mean calories consumed between or within groups
**Other results**	25% of customers reported that they eat at the restaurant at least once a week; health and taste top 2 reasons for selecting healthful items	Demographic variables had no effect on awareness	No information
**Sustainability**	Permanent implementation of some healthful foods	No information	No information
**Policy results and implications**	Evidence for feasibility of intervention in low-income setting	Moderate support for promotional campaigns as intervention strategy	None

**Table 3c:** Feasibility, Process, and Impact Results of Interventions on Prepared-Food Sources: Interventions Conducted in Small Local Restaurants^a^

Characteristic	Shape Up Somerville (32–36)	Smart Menu Program (37,38)	The Healthy Options Program (39–42)
**Feasibility and process results**	Low acceptability; medium reach	High acceptability; low feasibility; low reach; low operability	Moderate acceptability of promoted items; high fidelity; high feasibility
**Prepared-food source impact results**	4/10 Restaurants changed menus; 6/10 reported customers ordering from Shape Up Somerville options; 7/10 believed beneficial to participate; 7/10 were more aware of nutrition; 4/10 thought customers were more aware of nutrition	Fewer average calories, lower levels of fat and sodium per entrée sold	No significant change in ordering
**Consumer psychosocial impact results**	Not assessed at restaurant level	High level of awareness; no impact on knowledge reported	Moderate awareness
**Consumer behavioral impact results**	Not assessed at restaurant level	20.4% of customers reported ordering lower calories, 16.5% lower fat	1/3 of customers reported materials influenced ordering
**Other results**	Body mass index among children reduced by 0.1005	Higher entrée cost associated with more calories and fat consumed; consumers chose smaller, cheaper entrées	None
**Sustainability**	Low-medium: more than 50% of restaurants were noncompliant at follow-up	Medium: resource-intensive intervention.	High: materials stayed in place
**Policy results, implications**	Needed a stronger prepared-food source component	Success for calorie-labeling policy	Possibilities for combination with other intervention strategies

a Includes small, locally owned “mom-and-pop” establishments that include but are not limited to take-out and sit-down restaurants and restaurants that focused on specialty foods; it excludes chain restaurants.

**Table 3d:** Feasibility, Process, and Impact Results of Interventions on Prepared-Food Sources: Interventions Conducted in Mixed Types of Restaurants^a^

Characteristic	Healthy Howard Initiative (43)	Healthy Restaurant Program (44–46)	Winners Circle Healthy Dining Program (47–49)
**Feasibility and process results**	Moderate reach (currently on-going)	Increasing reach; improved ratings of participating restaurants	High feasibility; medium-low reach
**Prepared-food source impact results**	Not assessed (surveys not performed)	Increase in sales of promoted items; sodium and fat in foods significantly decreased	Not assessed
**Consumer psychosocial impact results**	Assessed but not reported	High customer acceptability	Low awareness
**Consumer behavioral impact results**	Assessed but not reported	Not assessed	Medium use of label for food choice
**Other results**	None	None	None
**Sustainability**	Low implementation cost	High: 85/96 of restaurants are maintaining program	Low-cost, easily implemented
**Policy results, implications**	Voluntary program	Ordinance for the Healthy Restaurant Program	No links with policy

a Includes restaurants (both local and chain) that did not share characteristics with other intervention restaurants or whose characteristics were not well defined.

Findings on process evaluation were more variable. Several interventions were public health interventions with open volunteer enrollment. Many of these interventions had low reach, when assessed by counting customers. We found that interventions had particular difficulty in recruiting restaurants, which was reflected in low reach among restaurants (37,38,47–49). One exception was Healthy Howard, which had moderate reach (43). At least 1 intervention (Shape Up Somerville [32–36]) experienced a decrease in participation over time, whereas others such as Healthy Howard (43) and Healthy Restaurant (44–46) experienced an increase. Only 2 interventions, Baltimore Healthy Carryouts (15–23) and the Healthy Options program (39–42), assessed fidelity; both demonstrated high fidelity. The level of acceptability was high among restaurant owners when the intervention was perceived as less burdensome (15–23,28,29,37–42) because of incentives such as free menu analyses, point-of-purchase materials, and media promotions.

#### Impact of interventions on prepared-food sources

Seven interventions assessed the impact of the intervention on prepared-food sources. Four of these 7 interventions (Baltimore Healthy Carryouts, Healthy Restaurant, Healthy Howard, and Shape Up Somerville) interviewed food-source owners to assess whether sales of healthful options increased. Four used data from food-source registers; 2 interventions collected sales receipts (Baltimore Healthy Carryouts [15–23] and Horgen and Brownell [27]). Three interventions — Healthy Howard (43), Healthy Restaurant (44–46), and Shape Up Somerville (32–36) — collected information other than sales data from the owner or manager of the prepared-food source, including information on nutrition awareness.

When sales were assessed, 5 interventions showed an increase in unit sales of promoted foods. Three interventions — Horgen and Brownell (27), Healthy Restaurant (44–46), and Baltimore Healthy Carryouts (15–23) — documented increases in total intervention-associated sales. This finding is best substantiated in Baltimore Healthy Carryouts (15–23), which also examined sales in a comparison group of prepared-food sources and demonstrated an increase in total revenue among the intervention group relative to the comparison group (17,20).

#### Impact of interventions on consumers

Eleven interventions collected information on the impact of the intervention on consumers (15–23,25–31,37–49). Six interventions collected data on psychosocial factors such as program awareness and nutrition knowledge (28–31,37–42,47–49). Seven interventions collected information on behavior, particularly food choices made at prepared-food sources and frequency of use of prepared-food sources (15–23,28–31,37–42,47–49).

Of the 6 interventions that reported an assessment of psychosocial factors among consumers, most (n = 4) found an increased awareness of the intervention and its goals. Of these, Coeur en Santé (29), TrEAT Yourself Well (30), and Healthy Restaurant (44–46) showed improvements in acceptability of healthful food among customers.

Among the 7 interventions that assessed the impact of the intervention on consumer food-related behaviors, only 5 interventions reported results. Four of the 5 interventions (Baltimore Healthy Carryouts [15–23], TrEAT Yourself Well [30], Smart Menu [37,38], and Winners Circle [47–49]) found an increase in the frequency of purchasing the more healthful food options promoted by the intervention.

## Discussion

This is the first systematic review of interventions in community-based prepared-food sources. Results are promising, showing that cost-effective methods (eg, labeling foods as healthful) may have a significant impact on prepared-food source sales and customer behavior. Most approaches centered on signage to promote existing healthful food choices. Several worked with kitchen staff to improve low-fat food preparation practices, and several conducted formal menu analyses to determine more healthful choices for promotion. These strategies, though resource intensive, show that it is important to engage prepared-food source staff in determining more healthful options.

Although interventions in prepared-food sources are increasingly being considered viable public health interventions, it is clear from this review that the evidence base for these interventions is deficient. Study designs tended to lack comparison groups, treatment conditions were not randomly assigned, and selection criteria were not readily apparent; these inadequacies cast doubt on the generalizability of findings.

Many of the interventions included in this review were not formal studies but rather certification or campaign programs operated by local health departments. These programs were voluntary, which may explain why they varied in levels of reach. Low reach may have been due to the actual burden placed on restaurant owners by the intervention or the lack of potential benefits perceived by them. Partnerships between local public health departments and academic institutions may help overcome some of these limitations through improved social marketing of program strategies and benefits. For example, local public health departments could initiate the intervention and manage recruitment while an academic institution partner conducts a full impact assessment, including psychosocial surveys and sales data collection.

We found no clear preference for a conceptual or behavior-change framework. A few interventions used social marketing approaches, but these interventions generally did not incorporate key social marketing components, such as formative research, audience segmentation, or targeting of messages (50). New frameworks for operationalizing prepared-food source interventions need to be developed; such frameworks should incorporate elements of theories on the food environment (51,52) and behavior change.

Although many interventions showed promising results, most lacked adequate measurement of the impact of the intervention on consumers. Of studies that examined consumer outcomes, most assessed only awareness of the health promotion campaign and food purchasing frequency. More sophisticated assessments — based on behavior-change theories — are needed to evaluate such outcomes as food purchasing patterns and dietary intake. 

Almost half of the interventions lacked formal formative research — a major omission, especially considering that food environment research is an emerging area of public health programming. Formative research involves qualitative and quantitative data collection, which aids in program development (53–56); many interventions in our study would have benefited from this kind of preliminary research. In addition, most interventions focused only on promoting existing healthful options at prepared-food sources. Only 1 intervention sought to introduce new, more healthful foods. Interventions need to pay more attention to actively changing the food environment in prepared-food sources by increasing the availability of healthful food options.

Another approach worth exploring is price changes. Two interventions showed positive results by reducing food prices (Baltimore Health Carryouts [15–23] and Horgen and Brownell [27]). Ample evidence supports the use of price reduction as a means of promoting more healthful options among consumers (57–59). Moreover, research on pricing may support the hypothesis that the increased prevalence of obesity may be attributed to greater consumption of soda and chips that have artificially low prices because of government subsidies for corn and soybeans (4,60,61). If nonhealthful and healthful food prices were comparable, consumers might be more likely to purchase healthful foods. Baltimore Healthy Carryouts demonstrated that reducing the price of healthful foods not only increased sales of healthful foods but also increased total carryout revenue (17,20).

Our findings parallel in many ways the findings of recent interventions in small retail food stores (14). Both types of interventions focused on modifying the food environment and promoting healthful choices through point-of-purchase materials. A key difference, however, was that most research on small food stores has focused on food deserts in primarily low-income racial/ethnic minority populations. Only a few of the interventions in our study took place in low-income settings. More carefully evaluated interventions in food sources in low-income settings are needed. The high rates of obesity and other chronic diseases are related not only to the availability of healthful choices in retail food stores but also to access to healthful foods in prepared-food sources (15,62–64). Although a recent longitudinal study (63) showed mixed results on the relationship between the availability of grocery stores and diet-related outcomes, it demonstrated that the availability of fast food was related to fast food consumption in a low-income population. Low-income populations tend to rely on local prepared-food sources because they often work more than one job and do not have time to cook at home (65). One study found that during the 2007–09 recession, the middle quintile of households (the middle 20% of the income distribution) cut spending on food away from home by 20%, whereas the lowest quintile of households cut such spending by 12%, suggesting a greater reliance on food away from home among lower quintile households (66). Improving the availability of healthful food in prepared-food sources may be an effective way to promote dietary improvement in low-income settings.

This systematic review has some limitations. Many of the interventions reviewed were implemented by health departments that lacked the resources to conduct comprehensive evaluations and publish the findings in peer-reviewed publications. Thus, we included findings from the gray literature. Because of the wide variability in measures used and in impact assessments, we were not able to develop summary estimates or compare measures or impacts directly among interventions. These limitations emphasize the need for standardization of measures used by interventions and the need for further reviews that assess different strategies (14).

Our review lays the groundwork for further exploration of strategies to increase more healthful food options in community-based prepared-food sources. Many interventions showed that changing the prepared-food environment may improve sales and awareness of more healthful foods and improve purchasing and consumption behaviors. Interventions can be strengthened through comprehensive formative research and quantitative assessments of process and impacts. With these additions, future studies will be able to assess the relative effectiveness of different strategies and create standards of practice in prepared-food sources.

## Appendix. Adjudication Chart for Study Review

**Table:**

Topic	Reviewer 1 long response	Reviewer 1 short summary response	Reviewer 2 long response	Reviewer 2 short summary response	Adjudication (as it appears in the final table)
Project name					

Data sources					

Target population (ethnicity, age group, geographic location, etc.)					

Model/Theory					

Goal or Purpose of the trial (increase availability, increase sales, modify consumer diet, etc.)					

Food (foods that were the focus of the intervention)					

Intervention strategies: signage					

Intervention strategies: availability of healthy foods					

Intervention strategies: pricing or cost					

Intervention strategies: community components					

Intervention strategies: other					

Study design					

Formative research					

Feasibility assessment (acceptability, operability, perceived sustainability)					

Process evaluation (how well the program was implemented according to plan)					

Prepared-food source impact measures					

Consumer impact measures (psychosocial, behavioral, health outcomes)					

Feasibility and process results					

Prepared-food source impact results					

Consumer psychosocial Impact results					

Consumer behavioral impact results					

Other results					

Sustainability					

Policy results, implications					
